# Risk of Fractures, Repeated Fractures and Osteoporotic Fractures among Patients with Hemophilia in Taiwan: A 14-Year Population-Based Cohort Study

**DOI:** 10.3390/ijerph20010525

**Published:** 2022-12-28

**Authors:** Yuan-Yi Pai, Jiaan-Der Wang, Hsin-En Ho, Yi-Jung Chou, Wen-Chao Ho, Wei-Cheng Chan, Wei-Min Chu, Yu-Tse Tsan

**Affiliations:** 1 Department of Family Medicine, Taichung Veterans General Hospital, Taichung 40705, Taiwan; 2 Division of Occupational Medicine, Department of Emergency Medicine, Taichung Veterans General Hospital, Taichung 40705, Taiwan; 3 Center for Rare Disease and Hemophilia, Department of Pediatrics, Taichung Veterans General Hospital, Taichung 40705, Taiwan; 4 Department of Family Medicine, Taichung Armed Force General Hospital, Taichung 411228, Taiwan; 5 Institute of Medicine, Chung Shan Medical University, Taichung 40201, Taiwan; 6 School of Medicine, National Defense Medical Center, Taipei 11490, Taiwan; 7 Division of Insurance Services, National Defense Medical Center, Taipei 11490, Taiwan; 8 Department of Public Health, China Medical University, Taichung 406040, Taiwan; 9 School of Medicine, National Yang Ming Chiao Tung University, Taipei 112304, Taiwan; 10 Department of Post-Baccalaureate Medicine, College of Medicine, National Chung Hsing University, Taichung 40227, Taiwan; 11 Education and Innovation Center for Geriatrics and Gerontology, National Center for Geriatrics and Gerontology, Moriokamachi 474-8511, Japan; 12 School of Medicine, Chung Shan Medical University, Taichung 40201, Taiwan

**Keywords:** hemophilia, fracture, osteoporosis, osteoporotic fracture

## Abstract

The world is aging, and hemophilia patients are as well. The association between patients with hemophilia (PWH) and low bone mineral density is clear. However, the incidence of fractures in patients with hemophilia is inconclusive, and no research has yet explored repeated fractures among PWH. In this study, we investigated the incidence of all-site fractures, repeated fractures and osteoporotic fractures amongst PWH. The study compared the incidence of all-site fractures, repeated fractures and osteoporotic fractures occurring in all PWH who were enrolled in Taiwan’s National Health Insurance Research Database between 1997 and 2013 with an age- and gender-matched group from the general population. Eight-hundred thirty-two PWH, along with 8320 members of the general population, were included in the final analysis. After multivariate COX regression analysis with an adjustment for confounding factors, it was found that PWH experienced a higher risk of osteoporotic fracture (HR: 1.25 with 95% CI of 1.03–2.52) but only saw a neutral effect with regards to both all-sites of fracture (HR: 1.00 with 95% CI of 0.92–1.09) and repeated fractures (HR: 1.01 with 95% CI of 0.92–1.10), when compared with the general population. This 14-year population-based cohort study showed that PWH had a higher risk of osteoporotic fracture, but that hemophilia only had a neutral effect in all-sites of fracture and repeated fractures. Screening, prevention and treatment for osteoporosis and further osteoporotic fractures among PWH, in order to improve quality of life and achieve healthy aging in this particular population, remain essential.

## 1. Introduction

Hemophilia A and B are both X-linked autosomal recessive disorders caused by defects in the factor VIII and factor IX genes. A deficiency in coagulation factors leads to different levels of bleeding tendency and any accompanying comorbidities, such as internal bleeding [1], arthropathy [2] and secondary osteoporosis [3]. In Taiwan, the annual crude incidence rates of hemophilia are at an average of 10.5 per 100,000 male births [4].

As the world is aging, so are hemophilia patients. The World Health Organization (WHO) defines healthy aging as a process of maintaining functional ability, ensuring the well-being in those at an older age [5]. Older patients with hemophilia (PWH) are developing comorbidities such as increasing rates of hypertension, obesity and diabetes, as well as cardiovascular disease (CVD), chronic kidney disease (CKD) and chronic joint arthropathy, which are all related to falls and fractures. By contributing to advances in the treatment of hemophilia, patients with hemophilia have experienced longer survival and, therefore, more opportunities to be confronted with those comorbidities [6,7]. 

In the past few decades, several research studies have suggested that there is an association between hemophilia and low bone mineral density [8,9]. However, it is difficult to ascribe hemophilia as the sole factor related to low bone mineral density. A single-center study in northern Greece revealed that hemophiliacs have an increased prevalence of low bone mineral density, and that bone mineral density is positively associated with hemophilia severity, any history of hepatitis virus C or human immunodeficiency virus and levels of physical activity [10]. Recent studies have focused on the role of antihemophilic factors and thrombin in bone remodeling. Despite the fact that there is lower bone mineral density in patients with hemophilia when compared with the general population, the etiology between hemophilia and low bone mineral density is still unclear [11].

People with low bone mineral density may be at a higher risk of experiencing osteoporotic fractures. Typically, osteoporotic fractures consist of hip, vertebral and wrist fractures. Few studies have focused on the relationship between fractures and hemophilia. The prevalence of fractures seen in previous studies was inconsistent, ranging from 4% to 37% [12]. With regard to the incidence of fractures in patients with hemophilia, a retrospective study in 2015 found a significantly higher incidence of fractures in PWH when compared with the general population, and that more osteoporotic fractures developed after middle age [13]. Another recent nationwide population-based comparative cohort study in Taiwan consisting of 75 patients with hemophilia and 300 matched control patients indicated that hemophilia may increase the risk of osteoporotic fractures, and that this risk was significantly higher for patients with hemophilia diagnosed over more than 5 years ago [14]. Although none of the studies reported on whether fractures affect the quality of life in patients with hemophilia, the expectation of a decreased quality of life in fracture patients with hemophilia may be widely accepted. Healthcare providers are now expected to pay more attention to the influence of fractures on patients with hemophilia.

However, to the best of our knowledge, there is no consensus on the incidence of fractures in patients with hemophilia, and no research has explored the incidence of repeated fractures among PWH. In patients with osteoporosis, fractures and repeated fractures are associated with a lower quality of life and higher mortality [15]. To refine the care of hemophilia patients, this study aims to assess the incidence rate of all-site fractures, repeated fractures and osteoporotic fractures in Taiwanese PWH with all ages and all levels of severity in order to clarify the interaction between hemophilia and fractures through a national-based retrospective cohort study.

## 2. Materials and Methods

We performed this retrospective cohort study using data from the Longitudinal Health Insurance Database (LHID) of the National Health Research Institutes (NHRI) of Taiwan. This National Health Insurance (NHI) program was adopted in 1995. Nearly all the residents of Taiwan are covered by this program, with approximately 23.75 million individuals currently in the registry. This LHID was set up for research purposes. All applications to access it must be from researchers or clinicians and are reviewed by experts to ensure the rationality of the request. The size of the LHID 2005 was restricted by NHRI and contains 1,000,000 subjects who were randomly sampled using a systematic sampling method from the 2005 registry of all insured individuals registered with the NHI. Though the sampling was done in 2005, the LHIDs contain data of sampled individuals from 1997 to the present day [16]. Under the maintenance of the Bureau of National Health Insurance (BNHI) and NHRI, the sample group showed no statistically significant differences in age, gender or healthcare costs when compared with all insured individuals [16]. The high-quality information maintained in these databases, including prescription use, diagnoses and hospitalization history, has been verified in previous epidemiological research [17,18]. The accuracy of diagnoses of major diseases listed in the LHID, such as acute coronary syndrome and stroke, has been validated [19,20].

In Taiwan, any severe illness which requires advanced health care with high treatment costs is defined as a catastrophic illness; hemophilia has been classified as such an illness. Patients confirmed as having a catastrophic illness are identified in the Registry of Catastrophic Illness Patient Database (RCIPD). In this study, the encrypted personal identification number found in the retrieved data was matched with the patient’s catastrophic illness certificate in the RCIPD to accurately identify hemophilia patients.

All male patients with either hemophilia A or B were selected by using the International Classification of Diseases Ninth Revision (ICD-9) codes 286.0 and 286.1 from the registration database and original claims data from the RCIPD during the period from 1 January 1997, to 31 December 2013. Data in National Health Insurance Research Database (NHIRD) patient files include the encrypted personal identification number, gender, date of birth, date of enrollment and medical records of each individual. Retracted hemophilic patients were matched at a ratio of 1:10 for the year of birth, gender and Charlson comorbidity index (CCI) with the general population in the LHID [21]. The CCI score consists of 19 medical conditions in a sum of the weights, which is a valid predictor of 10-year survival in patients with multiple comorbidities [22,23]. Any patients with osteoporotic fractures or fractures other than osteoporotic one which occurred prior to 1997 were eliminated from our study. We also excluded patients with the human immunodeficiency virus (HIV, ICD-9: 042) or Paget’s disease (ICD-9: 731), as those two diseases may affect fracture risk in patients with hemophilia. Both groups were observed within the same interval. The follow-up started on January 1, 1997, and ended either on 1 December 31, 2013, or if either of the pairings between the groups withdrew from the registry or died. The primary outcomes were fractures at any site, repeated fractures and osteoporotic fractures. Fracture at any site was defined as having had diagnosis of a fracture (ICD9:800–829, E887, A470-A476, A479) at least once as seen in the admission files, as well as 3 or more as seen in 1 year of the outpatient files during the study period [24]. Repeated fractures were defined as any new fracture at any site, with a diagnosis occurring more than 6 months after the first index fracture [25]. The osteoporotic fracture was defined as a fracture over the wrist, lumber spine or hip after 40 years of age (ICD-9:805,806,820,812–814), with at least 1 being seen in the admission files, as well as 3 or more being seen in 1 year of the outpatient files during the study period [26,27]. Any confounding factors, including hypertension, diabetes, chronic kidney disease, alcohol-related disease, thyrotoxicosis, dementia, obesity and osteoporosis were analyzed and compared among the PWH and control groups. We also recorded medications used for Hemophilia, such as Factor use, as a reference to the severity of the disease.

The Cox proportional hazards regression model was used to estimate the relationship of the different types of fractures between PWH and the general population. The hazard ratio (HR) for different types of fractures was then calculated, as well as 95% confidence intervals (CIs). A 0.05 of a 2-tailed *p* value was considered a significant result. All statistical analyses were conducted using SAS software (version 9.2; SAS Institute Inc., Cary, NC, USA).

## 3. Results

**Figure 1:**
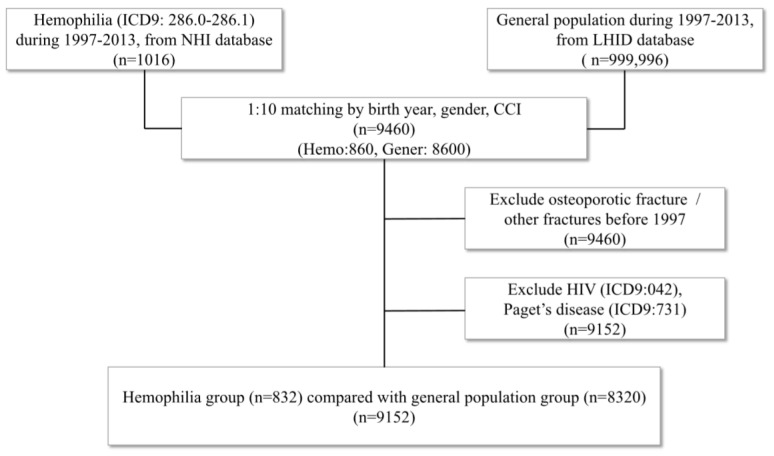
Flow chart of study design. (NHI = National Health Insurance; LHID = Longitudinal Health Insurance Database; CCI = Charlson Comorbidity Index; HIV = human immunodeficiency virus).

There were 1016 patients having the diagnosis of hemophilia during the period from 1 January 1997, to 31 December 2013, who were used for inclusion in this study. After performing matching for the year of birth, gender and CCI, we recruited 860 patients with hemophilia and 8600 people as the control group, for a total of 9460 participants. Amongst them, 308 patients were excluded due to a diagnosis of either HIV or Paget’s disease. The remaining 9152 participants who maintained the matching ratio of 1:10 were then entered into the analysis (Figure 1).

The characteristics of PWH and the general subjects are presented in Table 1. All the participants were male in both groups. Half of the patients with hemophilia, 416 patients out of 832, who were diagnosed with hemophilia were under 20 years of age. The residuals were given this diagnosis during adulthood. The 285 patients with hemophilia were diagnosed between the ages of 20–39, with 110 patients between 40–64 years of age. Only 21 patients, approximately 2.52% of the patients with hemophilia, were diagnosed after the age of 65. When discussing economic status, there was no difference in all income levels between PWH and the general population. In the hemophilia group, the prevalence of hypertension (14.66%), chronic kidney disease (1.8%), obesity (1.44%) and osteoporosis (5.89%) was significantly higher than that seen in the general population. The prevalence of those diseases in the general population was 9.78%, 0.96%, 0.44% and 1.48%, respectively. In contrast, the prevalence of diabetes, alcohol-related disease, thyrotoxicosis and dementia showed no difference between the two groups. Around 95% of PWH needed replacement therapy and only 6.73% of PWN was treated by bypassing agents in our study.

Analysis regarding primary outcomes for all-site fractures, repeated fractures and osteoporotic fractures is expressed in Table 2. For all-site fractures, 84 patients with hemophilia (10.1%) developed fractures during the study period, compared with 783 participants in the general population group (9.41%), indicating that there was no significant difference between the two groups (*p* = 0.52). There were 26 patients with hemophilia (3.10%) and 197 participants in the general population (2.36%) who developed repeated fractures with no significant difference between the groups (*p* = 0.18). Alternatively, a significant difference in osteoporotic fractures was noted between PWH and the general population (*p* = 0.03). Fourteen of the 832 patients with hemophilia (10.69%) experienced osteoporotic fractures compared to 76 of 8320 in the general population (5.80%).

**Figure 2:**
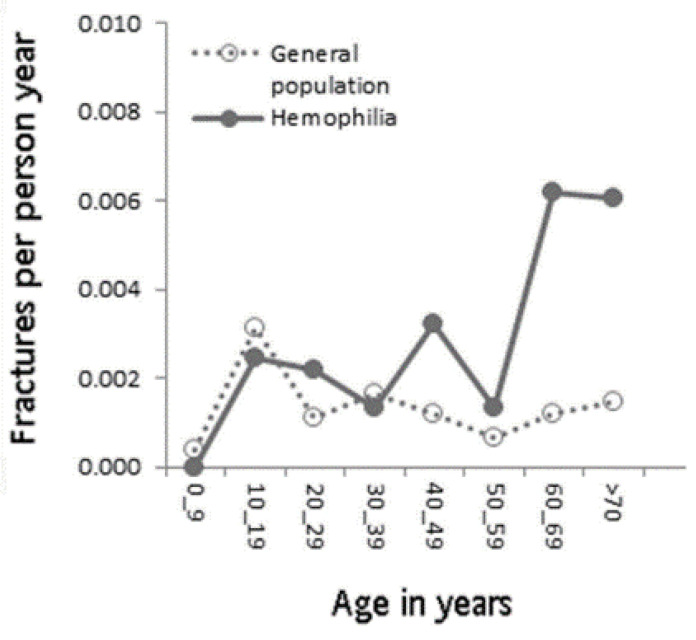
Incidence of all-site fractures per person-years at different ages among PWH and the matched control group.

Table 3 shows the hazard ratio of different types of fractures upon multivariate regression analysis. Confounding factors, including hypertension, chronic kidney disease and obesity were adjusted in Model 1. For all-site fractures and repeated fractures, the PWH group showed no increased risk when compared to the general population. For osteoporotic fractures in patients over 40 years of age, a significant increase in risk was found in PWH. Those effects seemed to be similar after adjustments. In Model 2, previous factors were adjusted, as well as osteoporosis. PWH had a lower risk towards developing all-site fractures or repeat fractures compared with the general population, (95% CI: 0.825–0.983, *p* = 0.019 and 95% CI: 0.825–0.980, *p* = 0.016 for all-site fractures and repeated fractures, respectively). On the contrary, no significant increase in risk was noted in the hemophilia group after adjustment with osteoporosis (95% CI: 0.939–1.387, *p* = 0.185). Furthermore, fracture incidence in hemophilia groups was higher than that seen in the general population, particularly for those aged 60 to 80. Fractures per person-years over different age groups are presented in Figure 2. In addition, the fracture incidence in patients with Hemophilia A and Hemophilia B was analyzed which showed that patients with Hemophilia A had a higher incidence rate of all site fractures after age more than 15, compared to patients with Hemophilia B (Supplementary Figure S1).

## 4. Discussion

The results of our study show that PWH experience a higher risk of osteoporotic fractures, but there is a neutral effect in all-site fractures and repeated fractures as seen in multivariate analysis. However, after adjustment for certain possible confounding factors, hemophilia became a protective factor against all-site fractures and repeated fractures.

In past decades, many studies involving meta-analyses have shown that low bone mineral density, regardless of adult or child patients with hemophilia, may be associated with osteoporotic fractures [8,9,10,28,29,30]. Two meta-analyses have demonstrated similar results showing reduced lumbar spine bone mineral density in adult and child patients with hemophilia, particularly in patients with severe hemophilia [8,9]. The most recent analysis included thirteen case-control studies and indicated that there was reduced lumbar spine bone mineral density (random effects standardized mean difference [95 % confidence interval (CI)] = −0.56 (−0.84, −0.28), between-study heterogeneity = 51 %) when compared with controls [9]. However, the exact molecular mechanisms of bone remodeling and bone loss in PWH are still not inclusive [11]. Few studies have been interested in the possibility that Factor VIII (FVIII) replacement therapy may reverse the phenotypes of the bone [31,32]. How FVIII affects bone health may be due to either a direct reaction or low FVIII effects, such as the missing interaction with vWF or decreased thrombin production. Though some studies have attempted to explain the mechanism, the discrepancies in data have led to difficulties in interpretation [31].

Low bone mineral density increases the risk of fractures. The relationship between low bone mineral density and PWH has been established, but fracture risk in PWH has been inconclusive. In our study, osteoporotic fracture risk and all-site fracture risk in patients with hemophilia was approximately 10%, which does not go against previous studies [12]. As previously mentioned, a retrospective comparative study involving 316 patients with hemophilia A and 66 with hemophilia B showed a significantly higher incidence of fractures in patients diagnosed with hemophilia (24.8 fractures per 1000 patient-years) when compared with the general population (9.6 fractures per 1000 patient-years). Additionally, the subgroup of those with mild to moderate hemophilia had a significantly decreased risk of fracture when compared to those with severe disease [13]. When compared to our study, we also disclosed a higher incidence in the hemophilia group, with the trend between ages and fracture risk in patients with hemophilia being similar to the previous study [13,33]. Fracture incidence in hemophilia groups was higher than that seen in the general population, particularly in the 60 to 80 age group, suggesting that fracture prevention in elderly PWH is very important. Previous literature has also emphasized the importance of appropriate management in the PWH group, suggesting the need for primary prevention of risk factors and close coordination between specialties [34].

However, to the best of our knowledge, no other studies have discussed repeated fracture risk. Additionally, after analysis with adjustment was performed, we found hemophilia to act as a protective factor for repeated fractures and all-site fractures. The possible reason for this may be because of patients decreasing their activities due to fear of falling or joint injury [35]. Although, a retrospective, descriptive research study performed in 2014 analyzed the occurrence of falls in community-dwelling moderate-to-severe hemophilia patients over the age of 40 and revealed that approximately one-third of participants had fallen in the past year. In that article, certain significant differences were found between non-fallers and multiple fallers. Multiple fallers had more joint prostheses, suffered more urinary incontinence, and had poorer balance or mobility as determined by the timed up-and-go test [36]. The risk factors noted in this study may be due to the presentation of aging. In our study, we included hemophilia patients of all ages regardless of the severity of their disease, with more younger patients being included in our study. Thus, we still suggest that a reduction in both exercise and activity during daily life may explain the protective effect seen in the hemophilia group regarding all-site fractures and repeated fractures. However, as current guidelines suggest that PWH should be encouraged to perform more physical activity [37], further research exploring physical activity and the incidence of all-site fractures and repeated fractures should be undertaken.

Despite all the efforts made in this study, there are still some limitations to note. First, although it is a population-based design, the external validity of the results should be carefully interpreted, particularly for non-Asian populations. Second, some confounders potentially related to osteoporosis in PWH such as hepatitis virus C infection, medications for osteoporosis, and steroid use were not adjusted and there were also no available records regarding physical exercise, diet habits, smoking, education level, family history, results of blood examinations or quantitative bone mineral density measurements in the NHIRD, therefore some residual confounders may exist. Third, since the NHIRD is an administrative database and not designed specifically for our research purpose, the severity of PWH could not be measured precisely through the serum factor activity. People with severe hemophilia may possess an increased risk of comorbidities, including all-site fractures and osteoporotic fractures. Further data collection and research are needed to figure out the underlying mechanism of fractures, repeated fractures, and osteoporotic fractures in the PWH.

## 5. Conclusions

This 14-year, population-based cohort study showed that PWH have a higher risk of osteoporotic fracture but experience a neutral effect with regards to all-site fractures and repeated fractures. As advancements in healthcare now allow for prolonged life for PWH, medical professionals caring for PWH should place greater emphasis on the screening, prevention and treatment of osteoporosis and possible future osteoporotic fractures among PWH patients, as this will no doubt improve patient quality of life and contribute to healthy aging within this particular population.

## Figures and Tables

**Table 1:** Characteristics of patients with hemophilia (PWH) and the matched control group.

Variable	Total	PWH	Matched Control Group	*p* Value ^†^
	(*n* = 9152)	(*n* = 832)	(*n* = 8320)	
Gender				-
Male	9152(100%)	832(100%)	8320(100%)	
Age (1997)				-
<20	4576(50.00%)	416(50.00%)	4160(50.00%)	
20–39	3135 (34.25%)	285(34.25%)	2850(34.25%)	
40–64	1210 (13.22%)	110(13.22%)	1110(13.22%)	
>=65	231(2.52%)	21(2.52%)	210(2.52%)	
Income (Missing = 4)				0.62
0	5038(55.07%)	439(53.02%)	4599(55.28%)	
1–15,840	1122(12.26%)	110(13.29%)	1012(12.16%)	
15,841–25,000	1901(20.78%)	178(21.50%)	1723(20.71%)	
>=25,001	1087(11.88%)	101(12.20%)	986(11.85%)	
Covariates				
Hypertension	936(10.23%)	122(14.66%)	814(9.78%)	<0.001
Diabetes	1169(13.68%)	146(18.79%)	1023(13.17%)	0.72
Chronic kidney disease	95(1.04%)	15(1.8%)	80(0.96%)	0.02
Alcohol related disease	154(1.68%)	19(2.28%)	135(1.62%)	0.16
Thyrotoxicosis	54(0.59%)	2(0.24%)	52(0.63%)	0.17
Dementia	121(1.32%)	12(1.44%)	109(1.31%)	0.75
Obesity	49(0.54%)	12(1.44%)	37(0.44%)	0.0002
Osteoporosis	172(1.88)	49(5.89%)	123(1.48%)	<0.001
Hip replacement	114(1.25)	88(10.58%)	26(0.31%)	<0.001
Drug use				
Bypassing	56(0.61%)	56(6.73%)		
Febia	33(0.36%)	33(3.97%)		
Novoseven	53(0.58%)	53(6.37%)		
Foactor use	792(8.65%)	792(95.20%)		
FVIII	636(6.95%)	636(76.44%)		
FIX	156(1.70%)	152(18.27%)		

^†^ ALL test used the χ^2^ test.

**Table 2:** Incidence of all-site fractures, repeated fractures and osteoporotic fractures among PWH and the matched control group.

	Total	PWH	Matched Control Group	*p* Value ^†^
	(*n* = 9152)	(*n* = 832)	(*n* = 8320)	
Fracture type				
Fracture (Y1)	867(9.47%)	84(10.1%)	783(9.41%)	0.52
Fractures (Y2)	223(2.42%)	26(3.10%)	197(2.36%)	0.18
Osteoporotic fracture (Y3)	90(6.25%)	14(10.69%)	76(5.80%)	0.03

Y1 means fracture at any site. Y2 means repeated fracture any site 6 months after index date of first fracture. Y3 means age > 40 Osteoporotic fracture. ^†^ ALL tests used the χ^2^ test.

**Table 3:** Multivariate COX regression analysis of all-site fractures, repeated fractures and osteoporotic fractures among PWH and the matched control group.

Fracture Type		Event	Crude HR	*p* Value	Adjusted HR	*p* Value
Y1		84	1.017 (0.933–1.109)	0.694		
	Model 1 ^†^				1.001 (0.918–1.091)	0.984
	Model 2 ^††^				0.9 (0.825–0.983)	0.019
Y2		26	1.023 (0.940–1.114)	0.595		
	Model 1 ^†^				1.006 (0.924–1.095)	0.898
	Model 2 ^††^				0.899 (0.825–0.980)	0.016
Y3		14	1.262 (1.039–1.533)	0.019		
	Model 1 ^†^				1.249 (1.028–1.517)	0.0255
	Model 2 ^††^				1.141 (0.939–1.387)	0.185

Y1 means fracture at any site. Y2 means repeated fracture at any site 6 months after index date of first fracture. Y3 means osteoporotic fracture. ^†^ Adjusted for Hypertension, Chronic kidney disease and Obesity in Model 1. ^††^ Adjusted for Hypertension, Chronic kidney disease, Osteoporosis and Obesity in Model 2.

## Data Availability

The datasets used and analyzed during the current study are not publicity available, but are available from the corresponding author upon reasonable request with the permission of Taichung Veterans General Hospital, Taiwan.

## Supplementary Materials

The following supporting information can be downloaded at: https://www.mdpi.com/article/10.3390/ijerph20010525/s1, Figure S1: Incidence of all-site fractures per person-years at different ages.

## References

[1] Berntorp E., Fischer K., Hart D.P., Mancuso M.E., Stephensen D., Shapiro A.D., Blanchette V. (2021). Haemophilia. Nat. Rev. Dis. Prim..

[2] Gualtierotti R., Solimeno L.P., Peyvandi F. (2021). Hemophilic arthropathy: Current knowledge and future perspectives. J. Thromb. Haemost..

[3] Rodriguez-Merchan E.C. (2022). Osteoporosis in hemophilia: What is its importance in clinical practice?. Expert Rev. Hematol..

[4] Chang C.Y., Yeh G.C., Lin S.Y., Tseng I.J., Tsai C.H., Lee Y.W. (2014). Trends in the epidemiology, diagnosed age and mortality rate of haemophiliacs in taiwan: A population-based study, 1997–2009. Haemophilia.

[5] Rudnicka E., Napierała P., Podfigurna A., Męczekalski B., Smolarczyk R., Grymowicz M. (2020). The world health organization (who) approach to healthy ageing. Maturitas.

[6] Angelini D., Konkle B.A., Sood S.L. (2016). Aging among persons with hemophilia: Contemporary concerns. Semin. Hematol..

[7] Chu W.M., Ho H.E., Wang J.D., Chan W.C., Liou Y.S., Ho W.C., Hu S.Y., Tsan Y.T. (2018). Risk of major comorbidities among workers with hemophilia: A 14-year population-based study. Medicine.

[8] Iorio A., Fabbriciani G., Marcucci M., Brozzetti M., Filipponi P. (2010). Bone mineral density in haemophilia patients. A meta-analysis. Thromb. Haemost..

[9] Paschou S.A., Anagnostis P., Karras S., Annweiler C., Vakalopoulou S., Garipidou V., Goulis D.G. (2014). Bone mineral density in men and children with haemophilia a and b: A systematic review and meta-analysis. Osteoporos. Int..

[10] Anagnostis P., Vakalopoulou S., Slavakis A., Charizopoulou M., Kazantzidou E., Chrysopoulou T., Vyzantiadis T.A., Moka E., Agapidou A., Garipidou V. (2012). Reduced bone mineral density in patients with haemophilia a and b in northern greece. Thromb. Haemost..

[11] Wang H., Bai X. (2021). Mechanisms of bone remodeling disorder in hemophilia. Semin. Thromb. Hemost..

[12] Anagnostis P., Karras S.N., Vakalopoulou S., Terpos E. (2016). Haemophilia and low bone mass. Ok, but what about fracture risk?. Haemophilia.

[13] Gay N.D., Lee S.C., Liel M.S., Sochacki P., Recht M., Taylor J.A. (2015). Increased fracture rates in people with haemophilia: A 10-year single institution retrospective analysis. Br. J. Haematol..

[14] Tuan S.H., Hu L.Y., Sun S.F., Huang W.Y., Chen G.B., Li M.H., Liou I.H. (2019). Risk of osteoporotic fractures as a consequence of haemophilia: A nationwide population-based cohort study. Haemophilia.

[15] Gold T., Williams S.A., Weiss R.J., Wang Y., Watkins C., Carroll J., Middleton C., Silverman S. (2019). Impact of fractures on quality of life in patients with osteoporosis: A us cross-sectional survey. J. Drug Assess..

[16] Hsieh C.Y., Su C.C., Shao S.C., Sung S.F., Lin S.J., Yang Y.H.K., Lai E.C.C. (2019). Taiwan’s national health insurance research database: Past and future. Clin. Epidemiol..

[17] Tsan Y.T., Lee C.H., Ho W.C., Lin M.H., Wang J.D., Chen P.C. (2013). Statins and the risk of hepatocellular carcinoma in patients with hepatitis c virus infection. J. Clin. Oncol..

[18] Wu C.Y., Chen Y.J., Ho H.J., Hsu Y.C., Kuo K.N., Wu M.S., Lin J.T. (2012). Association between nucleoside analogues and risk of hepatitis b virus–related hepatocellular carcinoma recurrence following liver resection. JAMA.

[19] Cheng C.L., Lee C.H., Chen P.S., Li Y.H., Lin S.J., Yang Y.H.K. (2014). Validation of acute myocardial infarction cases in the national health insurance research database in taiwan. J. Epidemiol..

[20] Cheng C.L., Kao Y.H.Y., Lin S.J., Lee C.H., Lai M.L. (2011). Validation of the national health insurance research database with ischemic stroke cases in taiwan. Pharmacoepidemiol. Drug Saf..

[21] Huang Y.C., Tsan Y.T., Chan W.C., Wang J.D., Chu W.M., Fu Y.C., Tong K.M., Lin C.H., Chang S.T., Hwang W.L. (2015). Incidence and survival of cancers among 1054 hemophilia patients: A nationwide and 14-year cohort study. Am. J. Hematol..

[22] Charlson M., Szatrowski T.P., Peterson J., Gold J. (1994). Validation of a combined comorbidity index. J. Clin. Epidemiol..

[23] Charlson M.E., Carrozzino D., Guidi J., Patierno C. (2022). Charlson comorbidity index: A critical review of clinimetric properties. Psychother. Psychosom..

[24] Lai S.W., Liao K.F., Lai H.C., Tsai P.Y., Lin C.L., Chen P.C., Sung F.C. (2013). Risk of major osteoporotic fracture after cardiovascular disease: A population-based cohort study in taiwan. J. Epidemiol..

[25] Hsiao P.C., Chen T.J., Li C.Y., Chu C.M., Su T.P., Wang S.H., Pan H.H., Wang K.Y. (2015). Risk factors and incidence of repeat osteoporotic fractures among the elderly in taiwan: A population-based cohort study. Medicine.

[26] Zhang H.W., Tsai Z.R., Chen K.T., Hsu S.L., Kuo Y.J., Lin Y.C., Huang S.W., Chen Y.P., Peng H.C., Tsai J.J. (2022). Enhanced risk of osteoporotic fracture in patients with sarcopenia: A national population-based study in taiwan. J. Pers. Med..

[27] Wang C.Y., Fu S.H., Yang R.S., Shen L.J., Wu F.L.L., Hsiao F.Y. (2017). Age- and gender-specific epidemiology, treatment patterns, and economic burden of osteoporosis and associated fracture in taiwan between 2009 and 2013. Arch. Osteoporos..

[28] Kempton C.L., Antun A., Antoniucci D.M., Carpenter W., Ribeiro M., Stein S., Slovensky L., Elon L. (2014). Bone density in haemophilia: A single institutional cross-sectional study. Haemophilia.

[29] Sossa Melo C.L., Wandurraga E.A., Peña A.M., Jimenez S.I., Salazar L.A., Ochoa M.E., Luna-Gonzalez M.L., Ortiz M.L., Morales K., Ayala-Castillo M. (2018). Low bone mineral density and associated factors in patients with haemophilia in colombia. Haemophilia.

[30] Sahin S., Sadri S., Baslar Z., Ar M.C. (2019). Osteoporosis in patients with hemophilia: Single-center results from a middle-income country. Clin. Appl. Thromb. Hemost..

[31] Gebetsberger J., Schirmer M., Wurzer W.J., Streif W. (2022). Low bone mineral density in hemophiliacs. Front. Med..

[32] Goldscheitter G., Recht M., Sochacki P., Manco-Johnson M., Taylor J.A. (2021). Biomarkers of bone disease in persons with haemophilia. Haemophilia.

[33] Johnell O., Kanis J. (2005). Epidemiology of osteoporotic fractures. Osteoporos. Int..

[34] Angelini D., Sood S.L. (2015). Managing older patients with hemophilia. Hematol. Am. Soc. Hematol. Educ. Prog..

[35] Taylor S., Room J., Barker K. (2020). Physical activity levels in men with haemophilia-a single centre uk survey. Haemophilia.

[36] Sammels M., Vandesande J., Vlaeyen E., Peerlinck K., Milisen K. (2014). Falling and fall risk factors in adults with haemophilia: An exploratory study. Haemophilia.

[37] De la Corte-Rodriguez H., Rodriguez-Merchan E.C., Alvarez-Roman M.T., Jiménez-Yuste V. (2012). Applying world health organization 2020 guidelines on physical activity and sedentary behavior to people with hemophilia. Expert Rev. Hematol..

