# Supplementary figures and images for: Risk of Fractures, Repeated Fractures and Osteoporotic Fractures among Patients with Hemophilia in Taiwan: A 14-Year Population-Based Cohort Study
Source: Int J Environ Res Public Health. 2022 Dec 28;20(1):525. doi: 10.3390/ijerph20010525 (PMC9819339; doi:10.3390/ijerph20010525)

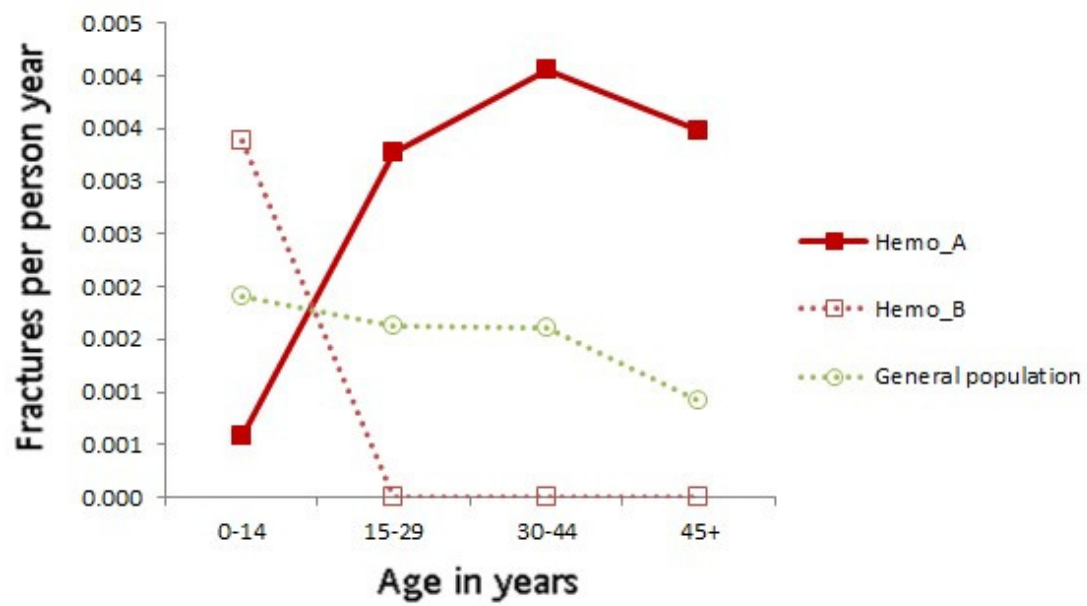

Figure S1. Age in years.

Supplement: Supplementary file 1 [file ijerph-20-00525-s001.zip › ijerph-2048655-supplementary.pdf]
